# An ITK implementation of a physics-based non-rigid registration method for brain deformation in image-guided neurosurgery

**DOI:** 10.3389/fninf.2014.00033

**Published:** 2014-04-07

**Authors:** Yixun Liu, Andriy Kot, Fotis Drakopoulos, Chengjun Yao, Andriy Fedorov, Andinet Enquobahrie, Olivier Clatz, Nikos P. Chrisochoides

**Affiliations:** ^1^CRTC Lab and Computer Science, Old Dominion UniversityNorfolk, VA, USA; ^2^Radiology and Imaging Sciences, National Institutes of HealthBethesda, MD, USA; ^3^Neurosurgery Department, Huashan HospitalShanghai, China; ^4^Radiology, Harvard Medical School, Brigham and Women's HospitalBoston, MA, USA; ^5^Kitware Inc.Clifton Park, NY, USA; ^6^Asclepios Research Laboratory, INRIA Sophia AntipolisSophia Antipolis Cedex, France

**Keywords:** image-guided neurosurgery, non-rigid registration, block matching, finite element, ITK, GPU

## Abstract

As part of the ITK v4 project efforts, we have developed ITK filters for physics-based non-rigid registration (PBNRR), which satisfies the following requirements: account for tissue properties in the registration, improve accuracy compared to rigid registration, and reduce execution time using GPU and multi-core accelerators. The implementation has three main components: (1) Feature Point Selection, (2) Block Matching (mapped to both multi-core and GPU processors), and (3) a Robust Finite Element Solver. The use of multi-core and GPU accelerators in ITK v4 provides substantial performance improvements. For example, for the non-rigid registration of brain MRIs, the performance of the block matching filter on average is about 10 times faster when 12 hyperthreaded multi-cores are used and about 83 times faster when the NVIDIA Tesla GPU is used in Dell Workstation.

## Introduction

Image-guided Neurosurgery (IGNS) is a system that can track in real-time the movement of the surgical tools in the patient space and report the movement to surgeons via the trajectory in the image space based on the established transform between the patient space and the image space. The transform is established before operations via routine point-based registration; however, during craniotomy, due to the drainage of the cerebrospinal fluid (CSF) and the operations, including tumor resection or retraction, the brain of the patient is deformed, which leads to inaccuracy in the preoperatively established transform. To recover the transform, the preoperatively acquired navigation image must be deformed accordingly. To achieve this end, a non-rigid registration can be used to align the preoperative image with the intra-operative modalities, such as Laser Range Scanning image, intra-operative ultrasound (iUS), or Magnetic Resonance Imaging (iMRI).

A clinically practical non-rigid registration method should consider the following factors: speed, robustness, and accuracy. The registration should be done within a short time period to provide timely responses to the surgeons. The registration results should not be susceptible to image intensity inhomogeneity and artifacts. The registration results should also realistically reflect the physical deformation of the tissue. Recently, Ur Rehman et al. (2008) presented a 3D non-rigid registration via optimal mass transport on the GPU. In this work, they presented a new computationally efficient numerical scheme for the minimizing flow approach for optimal mass transport and implemented this scheme on GPU. The results showed that this method is order of magnitude faster than previous work. Wang et al. (2010) presented a block matching based non-rigid registration method, in which the block matching was adapted and implemented on GPU. The resulting displacement vector field was smoothed by Gaussian and served to regularize the matching using normalized cross correlation. This method was applied to 4D lung CT images registration and planning CT and Daily Cone Bean CT registration. The landmark-based evaluation for both experiments showed the proposed GPU-based implementation achieved comparable registration accuracy, and compared to the CPU-based AtamaiWarp program, the GPU-based implementation is about 25 times faster.

In this paper, we parallelize a physics-based non-rigid registration (PBNRR) method (Clatz et al., 2005) based on our previous work (Chrisochoides et al., 2006; Liu et al., 2009) and integrate this fast, robust, and accurate non-rigid registration into the National Library of Medicine Insight Segmentation and Registration Toolkit (ITK). This work does not explicitly deal with tumor resection. If users want to use this method to find the deformation induced by tumor resection, they need to provide a tumor segmented mask image. The work specific for the tumor resection can be found in Drakopoulos et al. (2014). The registration method includes three components: feature point selection, block matching, and a robust Finite Element solver. All these components have been re-implemented in ITK in this work. ITK is a multi-platform open-source image analysis library serving many researchers and engineers worldwide. ITK collects many fundamental and cutting-edge image analysis algorithms, providing a platform for advanced product development. ITK has been in use for Visible Human project (The Visible Human Project^1^) and many commercial applications of the technology.

This paper makes the following contributions:

1. Four ITK filters, including one main filter and three sub-filters, are developed. The three sub-filters can be used, independent of the registration, for feature point selection, block matching, and a robust Finite Element solver, respectively. The main filter is used to combine these three sub-filters together to provide a user-friendly interface for non-rigid registration.
2. Both multi-core and GPU parallelization of block matching, a computationally intensive component of the registration, are developed to make optimal use of multi-core and GPU processors available to average computing platforms like desktops and laptops.

In the following sections, we first briefly describe the principle of the sequential non-rigid registration method and then present the details on the ITK implementation in section Materials and Methods. In the section Results, we present our experimental results of five clinical cases regarding performance and accuracy. After discussion of the correct usage of this method in clinical setting, we conclude our paper.

## Materials and methods

In this section, we first describe a PBNRR method including three critical components. We then present the ITK implementation of these components and a main ITK filter to connect these three components.

### Physics-based non-rigid registration method

Given preoperative MRI and intraoperative MRI, we aim to find the deformation between them and then deform the preoperative MRI according to the deformation. The main idea of the PBNRR method (Clatz et al., 2005) is to use the known displacement vector associated with sparse feature points in the brain to estimate the entire brain deformation with a brain biomechanical model. The biomechanical model is represented by a series of PDEs (Bathe, 1996), which describe the physical deformation of the brain. To find the numerical solution of the PDEs, Finite Element Method is employed by first discretizing the brain with a tetrahedron mesh and then using the displacements associated with the mesh nodes to represent the unknown continuous displacement field.

The registration method proposed in Clatz et al. (2005) includes three critical components:

1. Feature point detection: identify small image blocks that have rich structural information in the preoperative MRI.
2. Block matching: calculate displacement for each image block to generate a sparse deformation field.
3. Robust Finite Element solver: estimate entire brain deformation based on the sparse deformation field estimated above.

#### Feature point selection

The relevance of a displacement estimated with a block matching algorithm depends on the existence of highly discriminative structures within a block. We use the variance of the image intensity within the block region to measure its relevance and only select a fraction of all potential blocks based on a predefined parameter of the algorithm. To avoid redundancy produced by the overlapping of blocks (i.e., eliminate blocks which are too close to each other), a parameter of prohibited connectivity is used. Three connectivity patterns are supported in the ITK implementation: 6-connectivity, 18-connectivity, and 26-connectivity (see section Synthetic Data Evaluation). The prohibited connectivity allows the feature point selection to exclude neighboring feature points, which are connected to the current feature point via the prohibited connectivity. Thus, a higher connectivity pattern will exclude more neighboring feature points, therefore reducing the redundancy. To address the aperture problem (Poggio et al., 1985; Shimojo et al., 1989), the structural tensor of the block is calculated. The structural tensor reflects the distribution of the edge detections within the block, which will be incorporated into the Finite Element solver to make the estimated node displacement favor the reduction of the deviation along the direction orthogonal to the edge direction. To avoid finding false correspondence (e.g., the tumor resection cavity), the block selection utilizes a mask image when necessary to exclude certain portions of the image while searching for the feature points (e.g., in the case of tumor resection). The mask image is the segmentation result of the preoperative MRI. In this work, we use a Brain Extraction Tool (BET) (Smith, 2002) to extract the brain out of the skull and then manually refine the segmentation result. Users can use their own in-house segmentation tools or public tools to do the segmentation. After we get the mask image, we only perform feature point selection for blocks located in the mask image. There is no need to do segmentation for the intra-operative MRI.

#### Block matching

**Figure 1:**
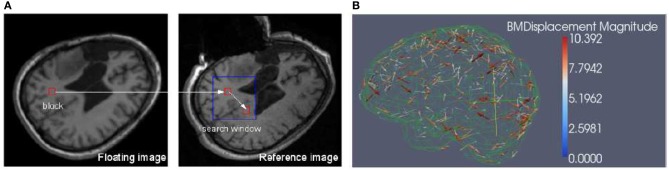
**(A)** Block matching. For a small block in the floating image, find its corresponding block in a predefined search window in the reference or fixed image, then the displacement associated with the block can be calculated. The block can be specified in both floating and reference images depending on the application. **(B)** Block matching results. The arrow points to the direction of the displacement and the color scale encodes the magnitude of the displacement. The metric is NCC and for clarity, only 1% of the dense displacement field is shown.

Block matching is a well-known technique widely used in motion coding, image processing and compression (Bierling, 1988; Stefano et al., 2007; Yuan and Shen, 2008). Block matching is based on the assumption that a complex non-rigid transformation can be approximated by point-wise translations of small image regions. Considering a block *B*(*O_k_*) in a floating image centered in *O*_*k*_ and a predefined search window *W*_*k*_ in a reference image, the block matching algorithm (as illustrated in Figure 1) searches for the position *O*_*m*_ in *W*_*k*_ that maximizes a similarity measure *M*. Similarity measures in this task include mean square difference of intensity (MSD), mutual information (MI), and normalized cross correlation (NCC).

(1) $$O_{m}=\underset{O_{i}\in W_{k}}{\arg\max}[M(B(O_{k}),\;B(O_{i}))]$$

We implemented NCC in itk::BlockMatchingImageFilter. Through exhaustive search, the location of the block that maximizes the similarity is obtained. By assembling the individual displacement vectors, one can create a sparse displacement field *D*, which is used by the solver to estimate the unknown displacement vector associated with the mesh nodes, i.e., the dense deformation field.

#### Finite element solver

The unknown dense displacement field *U* can be estimated by minimizing the energy function,

(2) $$W(U)=U^{T}KU+{\left(HU-D\right)}^{T}S(HU-D)$$

where the first regularization term describes the strain energy of a linear elastic biomechanical model, and the second term describes the error between the simulated displacements and the real displacements *D*. *U* is the unknown node displacement vector with a size of 3*n* · *n* is the number of nodes of the mesh. *K* is the mesh stiffness matrix of size 3 × 3*n* The assemblage of *K* has been well-documented in Bathe (1996). *H* is the linear interpolation matrix of size 3*p* × 3*n*, where *p* is the number of the registration points. *S* is the matching stiffness matrix of size 3 × 3*p*. The time for the assemblage of matrix *K* and *H* depends on the size of the mesh. For brain registration, the time is around several seconds for the mesh size satisfying the registration accuracy (Liu et al., 2009).

*K* describes the stiffness of the whole biomechanical system represented by a geometrical mesh and associated physical attributes, and *S* incorporates the stiffness, the balance parameter, matching confidence, and the local structure distribution of the feature points. *S* is a block-diagonal matrix whose 3 × 3 submatrix *S*_*k*_ is defined as $\lambda c_{k}\frac{n}{p}S_{k}^{\;avg}$, where λ is the balance parameter; *c*_*k*_ is the cross correlation computed from block matching for the *k*-th feature point. $\frac{n}{p}$ akes the matching term independent of the numbers of the vertices and the feature points. *S*^*avg*^_*k*_ is the average stiffness tensor for the *k*-th feature point (Clatz et al., 2005). *S*^*avg*^_*k*_ makes the registration point behavior like an elastic node of the finite element model, leading to the same measurement unit as the regularization term of function Equation (2).

The biomechanical finite element model is helpful in enforcing a realistic deformation on the brain. As a result, our priori knowledge about the stiffness of the intra-cranial structures can be introduced to the registration to estimate the deformation in the region far away from the feature points and regularize the deformation in the region near the feature points.

The sparse displacement field *D* is characterized by sparsity and outliers, which compromises the accuracy of the estimation of the dense deformation field *U*. To address the issue of sparsity of the deformation field, the estimation of *U* is regularized by a biomechanical model, which is capable of describing the physical deformation based on quite few data, i.e., the boundary condition. To make the estimation robust again outliers, *U* is estimated via Least Trimmed Squares (LTS) regression (Rousseeuw and Leroy, 1987). More specifically, we estimate *U* at each iteration, first without any outliers, then identify the points with larger error as outliers, and finally remove outliers from the data and re-estimate *U*. The above approximation formulation (Equation 2) performs well in the presence of outliers but suffers from an approximation error. Alternatively, solving the exact interpolation problem based on noisy data is not adequate. The robust solver developed in Clatz et al. (2005) can take advantage of both approximation and interpolation to iteratively estimate the deformation from the approximation to the interpolation while rejecting outliers. The gradual convergence to the interpolation solution is achieved through the use of an external force *F*, leading to the following iterative scheme,

(3) $$\begin{array}{l} F_{i}⇐KU_{i}\\ U_{i\;+\;1}⇐{\left[K+H^{T}SH\right]}^{-1}[H^{T}SD+F_{i}] \end{array}$$

The iterative scheme is derived as follows:

Taking derivative on both sides of Equation (2) and letting $\frac{\partial W}{\partial U}=0$, we obtain,

(4) $$[K+H^{T}SH]U=H^{T}SD$$

Equation (4) represents the balance between the internal mesh stress and the external force. Because the regularization term *U*^*T*^ *KU* (from the energy point of view) or the internal stress *KU* (from the force point of view) prevents the solution *U* from approaching the exact solution of the interpolation problem, we add an external force *F*_*i*_ ⇐ *KU*_*i*_ on the right side of Equation (4) at each iteration to balance the internal stress, which leads to the iterative scheme Equation (3).

### ITK implementation

**Figure 2:**
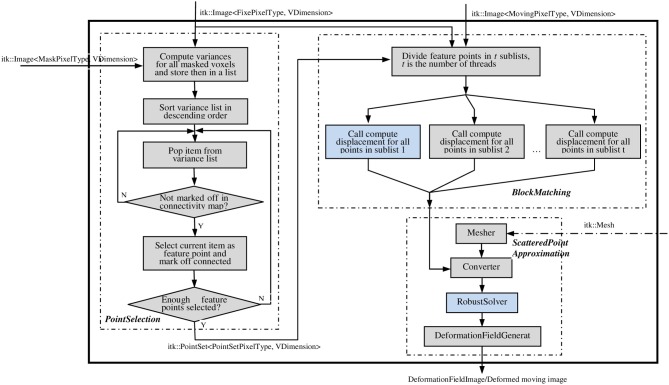
**The main filter PhysicsBasedNonRigidRegistrationMethod**. This filter takes the fixed, moving, and mask images as the necessary inputs (solid line); takes the mesh as the optional input (dashed line); and outputs a deformation fieldImage/deformed moving image. Figures 3A,B elaborate on the two highlighted compononets.

The ITK implementation of the PBNRR method contains three filters: MaskFeaturePointSelection, BlockMatchingImageFilter, and FEMScatteredDataPointSetToImage-Filter, which correspond to the above mentioned three components: feature point selection, block matching, and robust finite element solver, respectively. These three filters can function independently or be connected together to perform non-rigid registration. Block matching is parallelized using ITK v4 multithreading/GPU framework (OpenCL), for both multi-core and GPU, to accelerate the computation. The robust solver is enhanced to allow the accommodation of different geometry elements in dealing with linear elastic problems by simply providing appropriate mesh. To implement non-rigid registration and achieve ease-of-use, the three filters are combined into a single registration filter, PhysicsBasedNonRigidRegistrationMethod, as shown in Figure 2. This registration filter receives fixedImage, movingImage, maskImage, and an optional mesh as input and produces the dense deformation field as output. If users do not provide a mesh, a built-in hexahedral or rectangle mesh will be used.

#### ITK feature point selection filter

MaskFeaturePointSelectionFilter (see Figure 2) generates a list of feature points selected from a masked input image. It takes an Image and a mask Image as inputs and generates a PointSet of feature points as output. The feature points are physical centers of a small image blocks with higher variance. Optionally, a structure tensor may be computed and stored as a pixel value for each feature point. The following optional parameters can be set:

- NonConnectivity: defines connectivity pattern (VERTEX_CONNECTIVITY, EDGE_CONNECTIVITY or FACE_CONNECTIVITY) to a feature point. The default is VERTEX_CONNECTIVITY;
- BlockRadius: radius measured in voxels over which the variance is computed, its default value is 1;
- SelectFraction: fraction of points to select out of total eligible points, default is 0.05.

After the filter is created and inputs are set using SetInput and SetMaskImage, the Update method triggers calculation. After the Update, the method GetOutput returns a PointSet that contains coordinates of feature points as Point values and (optionally) structure tensors as Pixel values.

#### ITK block matching filter

**Figure 3:**
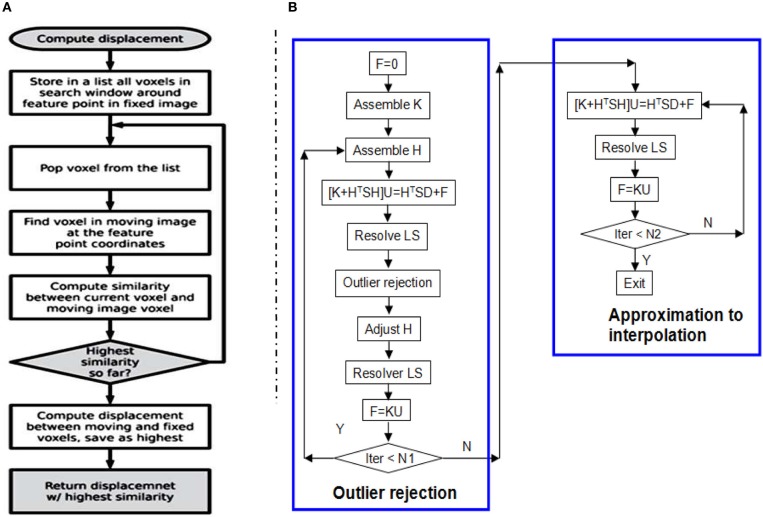
**(A)** The flow chart of one thread/kernel of block matching. **(B)** The flow chart of RobustSolver. RobustSolver includes two parts: outlier rejection and approximation to interpolation. Outlier rejection proceeds as a LTS regression (Liu et al., 2009): resolve *U* first, then detect outliers, remove outliers, and resolve *U* again. The *F* is used to reset the strain energy to enable the mesh to be deformed further. The difference between the two parts is the absence of outlier rejection in the approximation to interpolation part. RobustSolver supports both VNL solver and Itpack solver to resolve the linear system of equations. Compared to VNL solver, Itpacks runs faster, which is the default LS solver in RobustSolver.

BlockMatchingImageFilter (see Figure 2) computes displacements of given points from one image to another. This filter is parallelized using ITK multithreading and GPU. See Figure 3A for the flowchart of one thread/kernel. This filter takes fixed and moving Images, along with a PointSet of feature points, as inputs. The feature points are expected to lie at least SearchRadius + BlockRadius voxels from the image boundary. This is usually achieved by using an appropriate mask during selection of feature points. The default output (0) is a PointSet with displacement vector stored as the pixel value. Additional output (1) is a PointSet containing similarities, i.e., the NCC value. The number of points in the output PointSet is equal to the number of points in the input PointSet.

The following optional parameters can be set:

- BlockRadius: radius over which variance is computed, default is 1.
- SearchRadius: radius of the search window, default is 3.

After the filter is created and inputs are set using SetFixedImage, SetMovingImage, and SetFeaturePoints, the Update method triggers the calculation. The method GetDisplacements returns a PointSet that contains coordinates of feature points as Point values and displacement vectors as Pixel values. GetSimilarities returns a PointSet that contains coordinates of feature points as Point values and similarity values as Pixel values.

After Feature point selection and block matching, three point sets are available: feature point set with the structure tensor as the pixel value, block matching point set with the displacement as the pixel value, and the confidence point set with the similarity value as the pixel value. Block matching point set is a necessary input, and the other two are optional. These three point sets will be used by the FEMScatteredDataPointSetToImageFilter to perform scattered data approximation.

#### ITK scattered data approximation filter

The class RobustSolver implements the solver presented in section Finite Element Solver. This solver is a subclass of itk::Solver, which takes the FEMObject as input and output. FEMObject is an ITK data object to store all Finite Element related containers, such as mesh node container, mesh element container, landmark container, etc. We usually prefer a mesh and a feature point set as inputs and a deformation field image as the output. To use the RobustSolver with these natural inputs, we warp the RobustSolver in a FEMScatteredDataPointSetToImageFilter as shown in Figure 2. The FEMScatteredDataPointSet-ToImageFilter takes the mesh and feature point as inputs, converts these inputs into a FEMObject for the RobustSolver, and then generates a deformation field image based on the output FEMobject from the RobustSolver. Moreover, FEMScatteredDataPointSetToImageFilter provides a built-in 2D quadrilateral and 3D hexahedron mesh if the input mesh is not available.

Given a 2- or 3-D scattered and noisy point set, in which each point is associated with a 2-D or 3-D displacement, RobustSolver is able to approximate the data while rejecting outliers, advance toward interpolation, and ultimately output a deformed FEMObject, as outlined by the flowchart in Figure 3B. RobustSolver also takes into account two optional point sets: the confidence and the structural tensor. The confidence point set describes our confidence for each feature point using a value between 0 and 1 (0: not trustful, 1: completely trustful), which will make the solver behavior like a weighted Least Squares. The tensor point set describes the distribution of the edge direction within a small block surrounding the feature point, in order to avoid the aperture problem (Poggio et al., 1985; Shimojo et al., 1989).

## Results

In this section, we present our evaluation results for 2D synthetic and 3D MRI data. For the 2D synthetic experiment, we use the built-in rectangle mesh implemented in the FEMScatteredDataPointSetToImageFilter. The user needs to provide the spacing (physical unit) of the rectangle mesh, 20 mm in our experiment. The generation of the rectangle mesh is very straightforward. For the 3D MRI data, we use our in-house tetrahedron mesh generator presented in Liu et al. (2010). This mesh generation includes two steps: first produce a coarse Body-Centered Cubic (BCC) mesh based on the segmented mask image, and then compress the surface of the coarse BCC mesh to the boundary of the mask image. Users can refer to Liu et al. (2010) for details.

### Synthetic data evaluation

**Figure 4:**
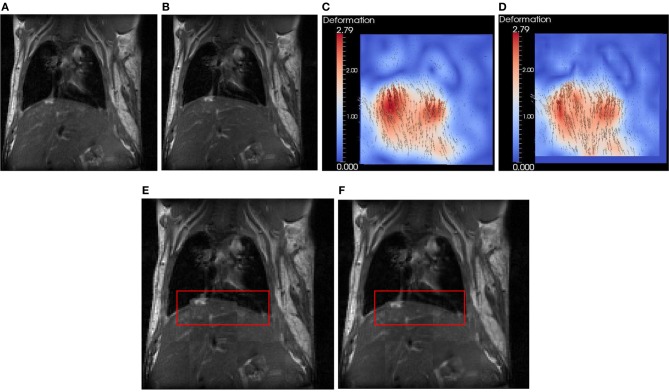
**Synthetic evaluation of FEMScatteredDataPointSetToImageFilter. (A)** the undeformed lung image, **(B)** the deformed lung image according to **(C)**, **(C)** the deformation field image (ground truth), **(D)** the estimated deformation field image, **(E)** the checkboard before regsitation, **(F)** the checkboard after registration. The red bounding box highlights the region with significant improvement of the accuracy after registration.

In this section, we use a lung image of a rat provided by ITK to evaluate FEMScatteredData-PointSetToImageFilter. The size of the lung image is 128 × 128, and the spacing is 1 × 1 mm^2^. This filter estimates the deformation field image based on the sparse deformation field. The approximated deformation field image can be further utlilized with itk::WarpImageFilter to produce an aligned image. To produce a sparse deformation field, we perform deformable registration on the lung images of a rat (see Figures 4A,B) using itk::BSplineDeformableTransform. The resulting deformation field image (ground truth) is shown in Figure 4C. We then perform edge detection in the fixed image (Figure 4A) to produce the edge image using the ITK canny edge detector. Finally, for all edge points, we perform interpolation in the deformation field to produce a sparse deformation field, which is represented by itk::PointSet. Since the edge detection is performed on the fixed image, the edge has the same origin as the fixed image. The displacement associated with the edge point can be directly obtained. Note that we focus on the assessment of the FEMScatteredDataPointSetToImageFilter in estimating the deformation field image from a sparse deformation field rather than on how to produce the input sparse deformation field. Users can use the tools they have to produce the sparse deformation field, not necessarily following the procedures presented in this paper. Figure 4D shows the estimated deformation field image, which is very similar with Figure 4C by visual inspection. Figures 4E,F show the checkerboard comparison before and after registration.

To quantitatively evaluate the accuracy, we calculated the error using ǁ *A* − *B* ǁ, in which *A* is the displacement in the estimated deformation field image, and *B* is the displacement in the ground truth. The mean ± SD, min and max errors are 0.7 ± 0.4, 0.0, and 2.1 mm, respectively.

### MRI evaluation

We conducted experiments on the registration between preoperative MRI and the intra-operative MRI (iMRI). The five datasets come from public cases from SPL of Harvard medical school (Talos and Archip, 2007). Table 1 lists the patient information including the gender, tumor location, and histopathology.

**Table 1:** **Patient information of five cases from SPL of Harvard medical school**.

**Case no**.	**Gender**	**Tumor location**	**Histopathology**
1	F	R occipital	Anaplastic oligodendroglioma WHO III/IV
2	F	L posterior temporal	Glioblastoma WHO IV
3	N/A	R frontal	Oligodendroglioma WHO II/IV
4	N/A	R occipital	N/A
5	F	R frontal	Oligoastrocytoma WHO II/IV

The MRI of the five public cases were acquired with a protocol of whole brain sagittal 3D-SPGR (slice thickness 1.3 mm, *TE*/*TR* = 6/35 ms, *FA* = 75°, *FOV* = 24 cm, matrix = 256 × 256) (Archip et al., 2007). In Table 2 we show the registration accuracy of the PBNRR filter for the five cases. As a measure of the registration accuracy, we used the one directional Hausdorff Distance (HD) as it is implemented in the vtkHausdorffDistancePointSetFilter. The HD(1 → 2) before PBNRR corresponds to the error between edge points in preoperative MRI and intra-operative MRI, while the HD(1 → 2) after PBNRR corresponds to the error between canny edge points in warped preoperative MRI and intra-operative MRI.

**Table 2:** **The registration accuracy evaluated by HD and landmarks for five cases**.

**Case no**.	**Before registration**	**PBNRR**	**BSpline NRR**	**PBNRR improvement**	**BSpline NRR improvement**
1	25.980 (12.874)	20.099 (8.522)	25.199 (10.853)	22.6 (33.8)	3.0 (15.7)
2	9.110 (7.490)	4.690 (2.073)	9.695 (6.539)	48.5 (72.3)	−6.4 (12.7)
3	9.433 (5.542)	5.385 (2.768)	8.124 (4.922)	42.9 (50.1)	13.9 (11.2)
4	9.695 (5.881)	7.000 (4.002)	9.434 (5.306)	27.8 (32.0)	2.7 (9.8)
5	6.708 (4.773)	4.123 (2.128)	7.141 (3.020)	38.5 (55.4)	−6.5 (36.7)

*The landmark evaluation results are listed in the parenthesis. A BSpline based non-rigid registration in 3DSlicer served as the comparison with the PBNRR. The parameters for PBNRR for all cases are: Block radius: [1,1,1], Window radius: [5,5,5], Selection fraction: 0.05, Rejection fraction: 0.25, Num of outlier rejection steps: 10, Num of approximation steps: 10, Young modulus: 694 Pa, Poisson's ratio: 0.45. The parameters for BSpline based registration are: Iteration: 20, Grid size: 18, Histogram bins: 100, Spatial samples: 50,000. Registration unit: mm, improvement unit: %*.

HD evaluation might be affected by outliers in the edge points, so we also performed landmark based evaluation. For each case, four landmarks were selected to calculate the accuracy of the method. These four landmarks include the morphologically special point in the vicinity of the resection region such as the vascular bifurcation points with obvious intensity enhancement, the frontal horn, and occipital horn of lateral ventricle and the choroid plexus of the triangular region of lateral ventricle. We selected four landmarks in the preoperative MRI, aligned preoperative MRI and iMRI, respectively, and there were totally 4 landmarks × 3 images × 5 patients = 60 landmarks selected. We use norm-2 of the displacement to calculate the error. Before registration, the error is calculated as ǁ *C* − *A* ǁ, and after registration the error is ǁ *C* − *B* ǁ, where A, B, and C represent the position of the landmark in the preoperative MRI, the aligned preoperative MRI, and the iMRI, respectively. For each case, the mean error serves as the evaluation of the method. The results are listed in Table 2. For case 2 and 5, it seems that BSpline based NRR degrades the accuracy regarding HD evaluation, but the landmark evaluation discloses that it might not be degradation but the influence of outliers.

We compared PBNRR with a popular BSpline based NRR (Cross-Correlation as the metric) in 3DSlicer (please see Table 2). For all five cases, PBNRR shows better results than BSpline based NRR regarding both HD and landmark evaluation.

**Figure 5:**
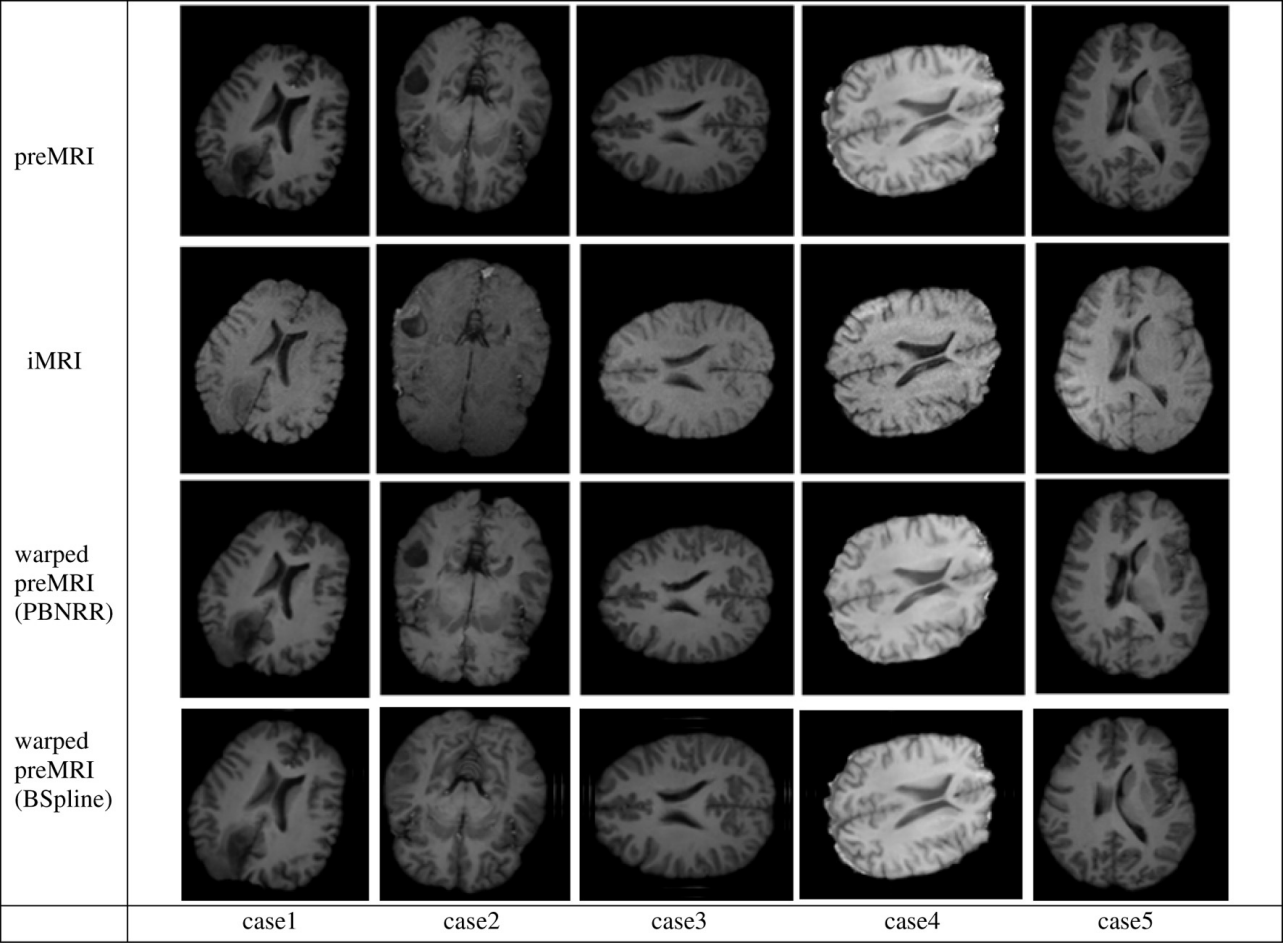
**The Qualitative results for the five cases of the PBNRR filter**. Each column corresponds to a different case, and each row from the top to the bottom: the preoperative MRI, the intra-operative MRI, and the warped preoperative MRI using PBNRR and the warped preoperative MRI using BSpline based NRR.

In Figure 5 we present the results of the PBNRR filter and the BSpline based registration for the same five cases we used throughout this evaluation.

In Table 3, we summarize the running time of the registration on three workstations. The running time includes the time for the PBNRR filter and the time for creating and writing the warped preoperative MRI, but does not include the time for generating the canny edge points and the calculation of the HD. Two Dell workstations are loacted in Old Dominion University (ODU) and one Cray XK7 workstation is loacated in National Center for Supercomputing Applications (NCSA). The running time of block matching of CPU and GPU is listed in Table 4.

**Table 3:** **The running time (second) of five cases for 3 workstations**.

**Case**	**Dell 1**			**Dell 2**			**Cray XK7**		
	**1 thread**	**12 threads**	**GPU**	**1 thread**	**12 threads**	**GPU**	**1 thread**	**16 threads**	**GPU**
1	54.53	37.73	33.50	54.40	37.83	33.62	136.72	116.25	105.46
2	60.36	41.49	37.60	59.72	41.44	37.57	155.70	126.70	120.95
3	52.19	35.79	32.25	52.45	35.90	32.38	131.51	111.05	102.54
4	65.14	44.60	40.24	65.54	45.60	40.75	173.15	145.60	135.79
5	52.36	35.44	32.20	52.50	35.59	32.55	129.22	111.17	101.42

*Dell 1: one Intel ® Core™ i7 CPU 260 @ 2.80 GHz, NVIDIA Quadro 6000 card, and 8 GB RAM. Dell 2: Intel(R) Xeon(R) CPU X5690 @ 3.47 GHz, Quadro 6000, and 96 GB RAM. Cray XK7: one AMD 6276 Interlagos Processor with 8 Bulldozer cores, 32 GB RAM, and NVIDIA Tesla K20X*.

**Table 4:** **Block matching running time (second) using CPU and GPU**.

**Case**	**Intel Intel(R) Xeon**		**Quadro 6000 GPU**	**AMD 6276**		**Tesla K20X GPU**
	**1 thread**	**12 threads**		**1 thread**	**16 threads**	
1	19.06	1.83	0.49	31.29	3.32	0.37
2	21.08	1.96	0.54	34.57	3.58	0.41
3	18.88	1.96	0.50	30.98	3.27	0.37
4	23.43	2.65	0.60	38.19	3.96	0.45
5	18.97	1.77	0.48	28.13	3.29	0.37
Average speedup		10.07	38.85		9.35	82.70

*The speedup is with respect to 1 thread*.

In the three components, the block matching and FEM solver dominate the calculation of PBNRR. In this work, we only present the parallelization of the block matching in ITK. The parallelization of the FEM solver using PETSc can be found in our previous work (Liu et al., 2009). Due to license issue of PETSc, we do not parallelize the FEM solver in ITK. Comparing column 12 threads and column GPU with column 1 thread, we find the acceleration is not as large as the number of cores. This can be explained by the Amdahl's law that the sequential fraction limits the bound of the acceleration.

## Discussion

In this section, we will discuss the issues on how to apply the PhysicsBasedNonRigid-RegistrationMethod to the registration of preoperative MRI and intra-operative MRI for IGNS. One issue is how to specify the fixed image and moving image. Our purpose is to align the preoperative MRI to the intra-operative MRI and then use the warped preoperative MRI to guide the surgery.Therefore, the preoperative MRI should be the moving image and the intra-operative MRI should be the fixed image. Another issue concerns which image can be used to build the physical model. The proposed PBNRR method relies on a physical model to estimate the entire brain deformation. Building this model requires brain segmentation and mesh generation. Usually, we can perform these operations on the fixed image and then use the DeformationField, pointing from the fixed image to the moving image, to get the warped moving image. However, when we perform the registration in IGNS, there is no a sufficient amount of time to allow us to perform brain segmentation and mesh generation on the fixed image (intra-operative MRI) in the operating room, so we need to preoperatively finish these operations on the preoperative MRI. In this way, the resulting deformationField points from the preoperative MRI to the intra-operative MRI. To get the warped preoperative MRI, we have to invert the deformation field. To get the warped preoperative MRI, we provide a method CreateDeformedImage in the PhysicsBased-NonRigidRegistrationMethod to faciliate the calculation of the deformed preoperative MRI.

## Conclusion

We present an ITK implementation of a PBNRR method. The three filters: MaskFeaturePointSelection, BlockMatchingImageFilter, and FEMScatteredDataPointSetToImage-Filter can be used separately or combined together to conduct registration. MaskFeaturePointSelection identifies small blocks with rich structure information. BlockMatchingImageFilter finds the displacement associated with these blocks in order to produce a sparse deformation field, which is used by FEMScatteredDataPointSetToImageFilter to find the deformation field image. For each block, MaskFeaturePointSelection stores the structure tensor, and BlockMatchingImageFilter stores the confidence, i.e., the cross-correlation value. Both structure tensor and confidence are incorporated into the FEMScatteredDataPointSetToImageFilter to deal with aperture problem. To reduce the computational time, block matching is parallelized on multi-core and GPU. Data from the experiments of five brain MRI demonstrate the effectiveness of the non-rigid registration method.

## Future work

Although a default rectilinear mesh is provided inside, we strongly suggest users providing an anatomically adapted mesh as the input for the PBNRR due to its advantages in the accurate description of the geometry of the object and a small number of mesh nodes (unknowns). In the future, we plan to provide a web-service for image-to-mesh conversion to generate the mesh of the images over the WEB. This service can maintain new functionality as we better understand the needs of the ITK community. Moreover, due to the influence of the outliers to the HD evaluation, we intend to use a modified HD method presented in Garlapati et al. (2012) and the landmark to do more rigorous evaluation. Also, we are collecting more clinical MRI data to increase the number of test cases.

### Conflict of interest statement

The authors declare that the research was conducted in the absence of any commercial or financial relationships that could be construed as a potential conflict of interest.

## Footnotes

^1^The Visible Human Project. Available online at: http://www.nlm.nih.gov/research/visible/visible_human.html
